# Supplementary material for: Piecing together the biogeographic history of Chenopodium vulvaria L. using botanical literature and collections
Source: PeerJ. 2015 Jan 8;3:e723. doi: 10.7717/peerj.723 (PMC4304866; doi:10.7717/peerj.723)
Supplement: File S1 — Citations of the sources of locations and habitat descriptions of Chenopodium vulvaria. [file peerj-03-723-s001.docx]

Adler W, Mrkvicka AC. 2003. Die Flora Wiens, gestern und heute. Die wildwachsenden Farn- und Blütenpflanzen in der Stadt Wien von der Mitte des 19. Jahrhunderts p.152. Wien: Naturhistorisches Museum.

Akel E, Ismail ID, Al–Chaabi S, Fuentes S. 2010. New Natural Weed Hosts of Sweet potato feathery mottle virus in Syria. *Arab Journal of Plant Protection* 28:96–100.

Akyol Y, Altan Y. 2013. Flora of Maldan village (Manisa). *C.B.U. Journal of Science*. 8.2:1–16.

Alabama Plant Atlas Editorial Committee. 2014. Alabama Plant Atlas. [S.M. Landry and K.N. Campbell (original application development), Florida Center for Community Design and Research. University of South Florida]. University of West Alabama, Livingston, Alabama. Available at http://www.floraofalabama.org/SpecimenDetails.aspx?PlantID=1503. (accessed 5 May 2014)

Allorge P, Denis M. 1923. Une excursion phytosociologique aux lacs de Biscarrosse (Landes). *Bulletin de la Société botanique de France* 70:693–717.

Andersson G, Berggren J, Hamner JW. 1914. *Stockholmstraktens växter*. p.52.

Anon.. 1855. Harefield Plants. *The Phytologist* 1: p.63.

Anon. 1861. Kentish Botany. Plants of Ham, Pegwell Bay, Sandown Castle, Oldhaven, Sandwich, etc. *The Phytologist* 5: p.213.

Anon. 1861. Plants of Plymouth. *The Phytologist* 5:368–371.

Anon. 1862. Kentish Botany: Romney Marsh, its general Aspect, Formation, and Vegetation. *The Phytologist* 6:129–140.

Anon. 1868. Bericht über die fünfte Versammlung des preussischen botanischen Vereins za Elbing am 11. Juni 1867. Schriften der Königlichen Physikalisch–Ökonomischen Gesellschaft zu Königsberg 9: p.12.

Anon. 1884. Excursions and Entertainment of the Botanists at Philadelphia. *Botanical Gazette* p.163.

Anon. 1895. Nederlandsche Botanische Vereeniging. *Nederlandsch kruidkundig archief* 6: p.78.

Anon. 1904. Herborisation des 25–26 Juin 1904 Aux environs de Thouars (Deux–Sèvres). *Bulletin de la Société botanique des Deux–Sèvres* 16:163–165.

Anon. 1906. Excursion botanique faite à Saint–Savin (Vienne). le 25 juin 1905. *Bulletin de la Société botanique des Deux–Sèvres* p.260.

Anon. 1920. O Herbário do Colégio de S. Fiel. *Boletim da Sociedade Broteriana* 28:123–150.

Anon. 1921. Notes and Exhibits. *Proceedings of the Linnean Society of New South Wales* 46: p.503.

Anon. 1990. *Directory of Wetlands of International Importance*. p.299. Ramsar Convention Bureau.

Arbost MJ. 1891. Liste méthodique des plantes phanérogames et cryptogames vasculaires observes par les membres de la société française de botanique dans le massif du Cantal. *Revue de botanique* 9:503–530.

Ascherson P, Graebner P. 1896. *Synopsis der mitteleuropaïschen flora*. Leipzig: W. Engelmann.

Babel B. 1859. Uebersicht der flora von Rue–Vorpommern und Rügen. *Archiv des Vereins der Freunde der Naturgeschichte in Mecklenburg* 13: p.66

Babington CC. 1851. Manual of British botany, containing the flowering plants and ferns. Arranged according to the natural orders. J. Van Voorst. London, UK.

Baguet C. 1876. *Annotations Nouvelles a la Flore de la Province de Brabant*. p.21. Brussels: Royal Botanical Society of Belgium.

Baguet C. 1883. *Nouvelles acquisitions pour la Flore Belge*. Brussels: Royal Botanical Society of Belgium.

Baker JG. 1866. Flora of Gloucestershire New Gloucestershire Plants, Collected By St. Brody. *Journal of botany, British and foreign*. 4:121–123.

Baker JG. 1886. North Yorkshire: studies of its botany, geology, climate, and physical geography. *The transactions of the Yorkshire naturalists' union* 12: p.222.

Baker JG. 1892. North Yorkshire: studies of its botany, geology, climate, and physical geography. *The transactions of the Yorkshire naturalists' union* 17: p.359.

Baker JG, Tate GR. 1868. A new flora of Northumberland and Durham, with sketches of its climate and physical geography. Transactions of the Natural History Society of Northumberland, Durham, and Newcastle-upon-Tyne.

Ball J. 1878. *Spicilegium florae Maroccanae*. London: Taylor and Francis.

Barceló y Combis F. 1870. *Flora de las islas Baleares, seguida de un diccionario de los nombres baleares, castellanos y botánicos, de las plantas espontáneas y de las cultivadas*. Palma: Pedro José Gelabert.

Basköse I, Dural H. 2011. The Flora of Hasan (Aksaray Region, Turkey) Mountain. *Biological Diversity and Conservation* 4(2):125–148.

Beck G. 1887. Flora von Südbosnien und der angrenzenden Hercegovina. *Annalen des Naturhistorischen Museums in Wien* 2: p.60.

Beckmannen JC, Schulze E. 1906. J. C. Beckman's Flora von Anhalt. *Zeitschrift fur Naturwissenschaften* 78: p.325.

Béguinot A. 1914. Flora della mura e della via di Padova. *Malpighia* 27:244–259.

Bentham G, Hooker J. 1892. *Handbook of the British flora*. London: L. Reeve & Co.

Bergeret JP. 1909. *Flore des Basses–Pyrénées*. p.245. Pau: J. Empérauger.

Bevis JF, Griffin WH. 1909. *The Flora of Woolwich and West Kent. A survey and record of Woolwich and West Kent* 31–230. Woolwich.

Bianchini A. 1909. Di alcuni nomi volgari di piante in Rieti. *Bollettino del naturalista* 29(3–4): p.109.

Bogenhard C. 1850. *Taschenbuch der Flora von Jena, oder, Systematische Aufzählung und Beschreibung aller in Ostthüringen wildwachsenden und kultivirten Phanerogamen und höheren Cryptogamen: mit besonderer Berücksichtigung ihres Vorkommens nebst einer Dorstellung der Vegetationsverhältnisse der bunten Sandstein-, Muschelkalk- und Keuperformation in mittleren Saal- und Ilmgebiete*. Leipzig: W. Engelmann.

Boissier E, Buser R. 1879. F*lora Orientalis : sive, Enumeratio plantarum in Oriente a Graecia et Aegypto ad Indiae fines hucusque observatarum IV*. Basileae: H. Georg. p.901.

Bonnet E. 1912. Enumération des plantes recueillies par M. Chudeau dans I'Ahaggar. *Bulletin du Muséum d'histoire naturelle* 18:513–516.

Boreau A. 1859. *Catalogue raisonné des plantes phanérogames qui croissent naturellement dans le département de Maine et Loire*. p.140. Angers: Cosnier et Lachèse.

Boswell Syme JT. 1868. *English botany*. London: Robert Hardwicke.

Brandes W. 1905. Zweiter Nachtrag zur Flora der Provinz Hannover. *Jahresbericht der Naturhistorischen Gesellschaft zu Hannover* 50–54:137–221.

Briard J. 1881. *Catalogue raisonné des plantes observées jusqu'à ce jour qui croissent naturellement dans le département de l'Aube*. p.195. Troyes: Dufour-Bouquot.

Briquet J, de Litardière R. 1910. *Prodrome de la flore corse: comprenant les résultats botaniques de six voyages exécutés en Corse sous les auspices de M. Emile Burnat*. 1: p.455. Genève: Georg & Co.

Bristol Naturalists' Society. 1881. *Flora of the Bristol coal-field*. Bristol: J. Fawn & Son.

Britten J. 1867. Notes on Buckinghamshire plants. *Quarterly Magazine of the High Wycombe Natural History Society* 1: p.172.

Bromfield WA. 1850. A Catalogue of the Plants growing wild in Hampshire, with occasional notes and observations on some of the more remarkable Species. *The Phytologist* 3:745–768.

Bromfield WA, Hooker WJ, Salter TB. 1856. *Flora vectensis*. London: William Pamplin.

Bubani P, Penzig O. 1897. *Flora Pyrenaea per ordines naturales gradatim digesta* 1: p.175.

Bucher CT. 1806. *Florae Dresdensis nomenclator*. Dresden: Walther.

Burk I. 1877. List of Plants Recently Collected on Ships' Ballast in the Neighborhood of Philadelphia. *Proceedings of the Academy of Natural Sciences of Philadelphia* 29:105–109.

C T. 1875. The Islands of Finistère. *The Gardeners' chronicle* 3: p.333.

Calflora: Information on California plants for education, research and conservation, with data contributed by public and private institutions and individuals, including the Consortium of California Herbaria. [web application]. 2014. Berkeley, California: The Calflora Database [a non-profit organization]. Available: http://www.calflora.org/ (accessed on 5 May 2014)

Candargy CA. 1889. *Flore de l'île de Lesbos*. p.30. Üster-Zürich: A. Diggelmann

Cannarella P. 1909. Flora Urbica Palermitana. *Bullettino della Società Botanica Italiana* 5:172–191.

Cariot A, Chirat du Vernay, LCA. 1860. *Etude des fleurs, botanique élémentaire, descriptive et usuelle*. Lyon: Girard et Josserand.

Casu A. 1905. Contribuzione allo studio della flora delle saline di Cagliari. *Annali di botanica* 2:403–432.

Chalon J. 1862. Que sont devenues nos Plantes Rares de 1862? *Notes pour les environs de Namur*. p.21.

Champion De Crespingny E. 1877. A new London flora; or, Handbook to the botanical localities of the metropolitan districts. Compiled from the latest authorities, and from personal observations.

Chenevard P. 1910. *Catalogue des Plantes Vasculaires du Tessin*. Genève: Kündig. p.180.

Civit E. 1916. Catàleg del Herbari Jiménez Munuera de la Junta de Ciències Naturals (i). *Anuari / Junta de Ciències Naturals* 1: p.272.

Clapham AR, Tutin TG, Warburg EF. 1957. *Flora of the British Isles*. Cambridge: Cambridge University Press.

Clarke Heslington T. 1892. Casuals near Ripon. *The Naturalist*. p.342.

Cooper D. 1836. *Flora metropolitana*. p.4.

Cortesi F. 1906. Illustrazione dell' Erbario Borgia. *Annali di botanica* 4: p.247.

Cosmo Melvill J. 1876. *The flora of Harrow*. London: Longmans, Green and Co.

Curtis W. 1777. *Flora Londinensis*. 5. London: William Curtis.

Dalibard TF, Déséglise A. 1749. Florae parisiensis prodromus. p.77. Paris: Durand & Pissor.

Dandois H. 1865. Additions à la Florule des environs de Nivelles. *Bulletin de la Société Royale de Botanique de Belgique*. 4(2):139–154.

Debeaux JO. 1894. *Flore de la Kabylie du Djurdjura*. Paris: P. Klincksieck.

de Capel Wise JR, Crane W, Linton WJ. 1867. *The New Forest: its history and its scenery*. p.290.

de Figueiredo JJ. 1825. *Flora pharmaceutica e alimentar portugueza*. p.91.

de Loynes M. 1900. Liste des plantes recueillies ou observées dans l'excursion du 1° Juillet. *Actes de la Société linnéenne de Bordeaux* 55: p.222.

de Mariz J. 1897. Subsidios para o estudo da flora portugueza: Chenopodiaceace Amarantaceas de Portugal. *Boletim da Sociedade Broteriana*. 14:175–208.

de Natale A, la Valva V. 2000. La flora di Napoli: i quartieri della città. *Webbia*. 54(2):271–375

Debeaux JO. 1894. *Flore de la Kabylie du Djurdjura, ou, Catalogue méthodique et raisonné de toutes les plantes vasculaires et spontanées observées jusq'à ce jour dans cette contrée*. Paris: Paul Klincksieck.

del Amo y Mora M. 1861. *Memoria premiada sobre la distribucion geografica de las plantas coneíferas, leguminosas, rosaceas, salsolaceas, amentáceas, coníferas y gramineas de la Península Ibérica*. Madrid : Aguado. p.340.

Dembitsky V, Shkrobb I, Hanusa LO. 2008. Ascaridole and related peroxides from the genus Chenopodium. *Biomed Pap Med Fac Univ Palacky Olomouc Czech Repub*. 152(2):209–215.

Dolenz V. 1910. Bericht der botanischen Sektion. *Mitteilungen des Naturwissenschaftlichen Vereines für Steiermark* 46: p.481.

Dörr E, Lippert W. 2001. *Flora des Allgaus*. Munchen: Bayerische Botanische Gesellschaft.

Farwell OA. 1901. A catalogue on the flora of Detroit. p.45.

Fiori A. 1923. *Nuova flora analitica d'Italia, contenente la descrizione delle piante vascolari indigene inselvatichite e largamente coltivate in Italia*. p.418.

Flower TB. 1870. The Flora of Wiltshire. *Wiltshire archaeological and natural history magazine*. 12(36):324–351.

Freitag H, Hedge IC, Jafri SMH, Kothe-Heinrich G, Omer S, Uotila P. 1997. Flora of Pakistan. http://www.efloras.org/florataxon.aspx?flora_id=5&taxon_id=242100047 (accessed 5 May 2014)

Re GF. 1881. La flora segusina di G. Francesco Re / riprodotta nel metodo naturale di De Candolle e comentata da Beniamino Caso. Torino: Per iniziativa e cura della Sezione di Susa del Club Alpino Italiano. p.288. http://biodiversitylibrary.org/page/10549921

Gillot F-X. 1904. Notes Botaniques. *Société d'histoire naturelle d'Autun* 17:116–166.

Godet CH. 1853. *Flora du Jura*. Neuchâtel: Godet. p.593.

Goiran A. 1893. Erborizzazioni estive ed autunnali attraverso i monti lessini veronesi. *Bullettino della Società Botanica Italiana* 8:433–439.

Goranova V, Vassilev K, Pedashenko H. 2013. Vascular flora of the Valley of Mesta River floristic region, SW Bulgaria. *Phytologia Balcanica* 19(1):89–114.

Gordon HD, Weaver J. 1877. The history of Harting, by the Rev. H.D. Gordon. With a chapter on the geology of the district, by the late Sir Roderick Impey Murchison, and some notice of its fauna and flora. p.483. London: W. Davy & son.

Gray SF. 1821. *A natural arrangement of British plants*. London: Baldwin, Cradock, and Joy.

Grubov VI. 2001. Key to the vascular plants of Mongolia. Enfield: Science publishers.

Hand R. 2006. Supplementary notes to the flora of Cyprus V. *Willdenowia* 36:761–809. doi:10.3372/wi.36.36211

Hansen AA. 1925. Recent Indiana weeds, 1925. *Proceedings of Indiana Academy of Science* 34: p.200.

Harding A, Outen A. 2009. Spring in North Cyprus (Naturetrek Tour Report). http://www.naturetrek.co.uk/reports_new/CYP01_report_090301_Spring_in_North_Cyprus.pdf

Hartman CJ. 1864. *Handbok i Skandinaviens flora : innefattande sveriges och norges växter, till och med mossorna*. p.172. Stockholm: Z. Haeggström.

Heller FX. 1810. *Flora Wirceburgensis*. p.278. Wirceburgi: Josephum Stahel. http://dx.doi.org/10.5962/bhl.title.6509

Hermann F. 1912. Ein botanischer Ausflug nach Majorka. *Verhandlungen des Botanischen Vereins für die Provinz Brandenburg* 54:239–257.

Heukels H. 1909. *De flora van Nederland*. 2: p.100.

Hilton T. 1898. *Abstracts of Papers Brighton and Hove Natural History and Philosophical Society. Botanical section*. p.45.

Hinterhuber R, Hinterhuber J. 1851. *Prodromus einer Flora des Kronlandes Salzburg und dessen angränzenden Ländertheilen*. p.182.

Hjelt H. 1904. Conspectus Florae Fennicae. *Acta Societatis pro Fauna et Flora Fennica* 30: p.129.

Höck F. 1899. Pflanzengeographie. Just's botanischer jahresbericht. *Systematisch geordnetes repertorium der botanischen literatur aller länder*. 27(1): p. 312.

Hooker WJ. 1821. *Flora scotica*. Edinburgh: A. Constable.

Hruby J. 1914. *Die Ostsudeten; eine floristische Skizze*. p.124. Brünn: Landesdurch-forschungs-Komemission.

Ilse H. 1866. *Flora von Mittelthueringen*. Erfurt.

Jafri SMH, Rateeb FB. 1978. Chenopodiaceae. *Flora of Libya* p.15.

Johns CA, Elliott C. 1911 . *Flowers of the field*. London: G. Routledge.

Johnson T. 1632. *Descriptio itineris plantarum investigationis ergo suscepti, in agrum cantianum*. London: T. Cotes.

Jorden G. 1855. Some account of the Botany of Wyre Forest and surrounding parts of the Country. *The Phytologist* 1: p.361.

Kandemir A, Coskunçelebi K, Beyazoglu O. 2000. A7 ve A8 Kareleri icin yeni floristik kayitlar. *Ot Sistematik Botanik Dergisi* 7(2):111–125.

Keddie W. 1844. Account of a Botanical Excursion to the Bass Rock, in July, 1844. *The Phytologist* 2: p.244.

Kitamura S. 1960. *Flora of Afghanistan*. p.100. Kyoto : The Committee of the Kyoto University Scientific Expedition to the Karakoram and Hindukush, Kyoto University

Kitchener G. 2014. Kent Rare Plant Register Draft species accounts C (second part: Ce-Cy). Available at http://bsbi.org.uk/rare_plant_register_accounts_C_-Ce-Cy-_Feb_2014.pdf (accessed 5 May 2014)

Kloos AW. 1915. Aanwinsten van de Nederlandsche Flora in 1915. *Nederlandsch kruidkundig archief* p. 194.

Klotz JPJ. 1875. Compte–rendu de la XIVe herborisation générale de la Société royale de Botanique de Belgique (1875). *Bulletin de la Société royale de botanique de Belgique* 3:248.

Koch KHE. 1839. *Das natürliche System des Pflanzenreiches nachgewiesen in der Flora von Jena*. p.130. Jena: Hochhausen.

Koch WDJ. 1856. *Taschenbuch der deutschen und schweizer Flora*. Leipzig: Gebhardt und Reisland.

Krasan F. 1918. Ergänzungen und Berichtigungen zu den älteren Angaben über das Vorkommen steirischer Pflanzenarten. *Mitteilungen des Naturwissenschaftlichen Vereines für Steiermark* 36:43160.

Krause EHL. 1915. Die nelken– und meldenartigen Gewächse Elsaß–Lothringens. Beihefte zum botanischen Centralblatt. *Erste Abteilung, Anatomie, Histologie, Morphologie und Physiologie der Pflanzen* 33:441–500.

Krombach JHW. 1875. *Flore du grand-duché de Luxembourg*. Luxembourg: Joris.

Laasimer L, Kuusk V, Tabaka L, Lekavicius A. 1993. *Flora of the Baltic Countries 1*. Tartu: Estonian Academy of Sciences. p.210.

Lajos S. 1886. Enumeratio Florae Transsilvanicae Vesculosae Critica. Budapest: Kiadja a Kir. Magyar Természettudományi Társulat. http://biodiversitylibrary.org/page/10524603

Lange J. 1856. *Haandbog i den danske flora*. p.185. Kjøbenhavn: C.A. Reitzels forlag.

Langethal CE. 1868. Beschreibung der Gewächse Deutschlands, nach ihrer natürlichen Familien und ihrer Bedeutung für die Landwirtschaft. Jena: Mauke.

Le Gall M. 1852. *Flore du Morbihan*. p.488. Vannes: J.-M. Galles.

Lefèvre E. 1866. Botanique du département d'Eure–et–Loir. Statistique scientifique du département d'Eure–et–Loir. p.164. Chartres: Petrot-Garnier.

Lejeune ALS, Courtois RJ. 1828. *Compendium florae Belgicae*. Leodii: P.J. Collardin.

Léveillé H. 1897. *Supplément à la flore de la Mayenne*. p.128. Le Mans: Ed. Monnoyer.

Lindemann E. 1867. Florim Elisabethgradems. Assitamentum ad Floram Chersonensem . *Bulletin de la Société impériale des naturalistes de Moscou* 40:297–379.

Lindley J. 1849. *Medical and economical botany*. London: Bradbury & Evans.

Linton WR. 1903. *Flora of Derbyshire*. p.249. London: Bemrose & Sons Ltd.

Löhr MJ. 1844. *Taschenbuch der Flora von Trier und Luxemburg*. Trier: C. Troschel.

Long AG. 1969. Natural History Observations during 1968. *History of the Berwickshire Naturalists' Club* 38: p.61.

Losa M, Montserrat P. 1951. Aportacion al conoclmiento de la flora de Andorra. Primer congreso internacional del Pirineo del Instituto de Estudios Pirenaicos 5–184.

Jüngst LV. 1837. *Flora von Bielefeld, zugleich die Standorte der seltneren Pflanzen im übrigen Westfalen*. p.90. Bielefeld: A. Helmich. http://biodiversitylibrary.org/page/6084496

Mabille MP. 1864. Des Plantes qui croissent autour de Dinan et de la Saint–Malo. *Actes de la Société linnéenne de Bordeaux* 25:489–656.

Macchiati L. 1888. Prima contribuzione alla Flora del Viterbese. *Atti della Società dei naturalisti e matematici di Modena* 3(7): p.44.

Maire RCJE. 1962. *Flore de l'Afrique du Nord*. Paris: Paul Lechevalier.

Maluquer Y, Nicolau S. 1901. Plantes més Notables dúna excurció a Montcada. *Butlletí de la Institució Catalana d'Història Natural* 3:13–15.

Maly JK. 1863. *Systematische Beschreibung der in Österreich wildwachsenden und kultivirten Medicinal-Pflanzen*. Wien: Wilhelm Braumüller.

Marcelo Rivas Mateos D. 1899. Flora de la Provincia de Cáceres. *Anales de la Sociedad Española de Historia Natural*. 28: p.153.

Marchesetti C. 1890. *Atti del museo civico di storia naturale di Trieste*. Trieste: Tipografia del Lloyd.

Margot H, Reuter FG. 1841. D´une Flore de l´ile de Zante. *Memoires de la Société de physique et d'histoire naturelle de Genève* 9.

Margelo Rivas Mateos D. 1931. *Flora de la Provincia de Caceres*. Serradilla: Sanchez Rodrigo. p.99.

Martersteck JC. 1792. *Bonnischer Flora oder, Verzeichniss aller hier wild– und frei–wachsenden Arznei–Pflanzen*. p.92. Bonn: Abshoven. http://dx.doi.org/10.5962/bhl.title.6151

Maw G. 1853. Notes on the Rarer Plants occurring in the Neighbourhood of the Estuary of the Taw and Torridge, North Devon. *The Phytologist* 4:785–795.

Melvill JC, Hind WM. 1876. *The flora of Harrow*. Harrow: Green and Co.

Mercadé A, Vigo J, Rull V, Vegas–Vilarrúbia T, Garcés S, Lara, A, Cañellas–Boltà N. 2013. Vegetation and landscape around Lake Montcortès (Catalan pre–Pyrenees) as a tool for palaeoecological studies of lake sediments. *Collectanea Botanica* 32:87–101. 10.3989/collectbot.2013.v32.008

Migout A. 1866. Flore du département de l'Allier. p.255. Moulins: Ducroux et Dulac.

Mochnacký S. 2012. Occurrence and distribution of Chenopodium vulvaria L. in Kosice city, Slovakia. Thaiszia, *Journal of Botany* 22(2):191–195.

Mohr C. 1901. Plant Life of Alabama. *Contributions from the United States National Herbarium* 6: p.488.

Mouterde P. 1966. Nouvelle Flore du Liban et de la Syrie. p.409.

Murr J. 1903. Agnoszierte Chenopodien. Allgemeine botanische Zeitschrift für Systematik, Floristik, Pflanzengeographie etc. 9: p.112.

Naveh Z. 1967. *Mediterranean Ecosystems and Vegetation Types in California and Israel*. Ecology. 48(3):445–459.

Neilreich A. 1868. *Flora von Wien; eine Aufzählung der in den Umgebungen Wiens wild wachsenden oder im grossen gebauten Gefässpflanzen, nebst einer pflanzen–geografischen Uebersicht*. Wien: Friedrich Beck. p.191.

Noulet JB. 1855. *Flore analytique de Toulouse et de ses environs*. Toulouse: Librairie centrale. p.133.

Presl JS. 1846. *Weobecný rostlinopis ili Popsani rostlin we welikém ohledu uitenych a kodliwých*. Czech: W. Praze.

Pries–Sehwerin K. 1906. Beiträge zur Flora von Cüstrin. *Verhandlungen des Botanischen Vereins für die Provinz Brandenburg* 48:107–113.

Printz H. 1921. The vegetation of the Siberian–Mongolian frontiers. *Contributiones ad floram Asiae interioris pertinentes* 3: p.209.

Pitard CJ, Proust L. 1908. *Les îles Canaries. Flore de l'archipel*. Paris: Paul Klincksieck.

Provost M. 1998. *Flore vasculaire de Basse-Normandie*. Caen: Presses Universitaires de Caen.

Puel T. 1852. *Catalogue des plantes qui croissent dans le département du Lot*. p.73. Cahors: J.P. Combarieu.

Rabenhorst L. 1850. Vorläufiger botauischer Bericht über meine Reise (durch die östlichen und stüdlichen Provinzen Italiens. *Flora oder Botanische Zeitung* 33:322–325.

Radde G. 1886. *Die Fauna und Flora des südwestlichen Caspi–Gebietes : Wissenschaftliche Beiträge zu den Reisen an der Persisch–Russischen Grenze*. Leipzig: F.A. Brockhaus.

Ravin E. 1861. Catalogue raisonné des plantes du département de l'Yonne. p. 203. Auxerre: Perriquet et Rouillé.

Rechinger KH. 1964. *Flora of Lowland Iraq*. p.185. Weinheim: J. Cramer.

Rechinger KH. 1977. Plants of the Touran Protected Area, Iran. *Iranian Journal of Botany* 1(2):155–180.

Reichenbach HGL. 1844. *Flora Saxonica. Die Flora von Sachsen, ein botanisches Excursionsbuch*. Dresden: Arnoldischen Buchhandlung.

Reichenbach HGL. 1850. *Icones florae Germanicae et Helveticae*. Lipsiae: F. Hofmeister.

Renger G. 1768. Die um Danzig wildwachsende Pflanzen nach ihren Geschlechtstheilen geordnet. p.94. Danzig: D.L. Wedel.

Reuter GF. 1832. Catalogue détaillé des plantes vasculaires qui croissent naturellement aux environs de Genève : avec l'indication des localités et de l'époque de la floraison. p.89. Genève: A. Cherbuliez.

Rieber X. 1897. Beiträge zur württembergisehen Flora. *Jahreshefte des Vereins für vaterländische Naturkunde in Württemberg* 53:139–141.

Riomet B. 1891. Flore de la Thiérache et d´une partie du Laonnois. *Revue de botanique* 10:35–163.

Rion C. 1872. *Guide du Botaniste en Valais*. p.177. Sion: A. Galerini.

Ritter Beck von Mannagetta G. 1890. Flora von Nieder–Österreich. p.330. Wein: Carl Gerold’s Sohn.

Robolsky H. 1843. *Flora der Umgegend von Neuhaldensleben*. Neuhaldensleben.

Röhling JC, Koch WDJ, Mertens FC. . 1823. *J.C. Röhlings Deutschlands Flora*. Frankfurt am Main: F. Wilmans.

Rohrer R., Mayer A. 1835. *Vorarbeiten zu einer Flora des Mährischen Gouvernements : oder systematisches Verzeichniss aller in Mähren und in dem K. K. Östr. Antheile Schlesiens wildwachsenden bis jetzt entdeckten phaenerogamen Pflanzen* p.58. Brünn: Rudolph Rohrer.

Rossetti C. 1893. Nuova contribuzione alla flora vascolare della Toscana. *Atti della Società toscana di scienze naturali, residente in Pisa. Memorie* 12:181–221.

Rossi L. 1915. Floristicka istrazivanja po jugoistocnoj hrvatskoj. p.22. U Zagrebu: Tisak Kr. Zemaljske Tiskare.

Rossi L. 1924. Grada za floru Juzne Hrvatske. *Prirodoslovna istrazivanja* 15: p.53.

Rothmaler W. 2005. *Exkursionsflora von Deutschland*. Munchen: Elsevier.

Rousseau MP. 1899. Catalogue des plantes vasculaires spontanées de I'île de Ré et des plantes qui y sont plus communément cultivées. *Bulletin de la Société des sciences naturelles de l'Ouest de la France* 9: p.184.

Sabransky H. 1908. Beiträge zur Flora der Oststeiermark. Verhandlungen des Botanischen *Vereins für die Provinz Brandenburg* 58: p.73.

Salmon JD. 1846. An Outline of the Flora of the Neighbourhood of Godalming, in the County of Surrey ; with brief Notices of the Geological Features of the District. *The Phytologist* 2: p.452.

Sanguinetti P. 1864. Florae Romanae prodromus alter : exhibens plantas vasculares circa Romam in Cisapenninis pontificiae dictionis provinciis in Umbria et Piceno sponte venientes: secundum sexuale systema dispositas p.213. Rome: Bonarum Artium.

Sauzé JC, Maillard PN. 1844. Flore du département des Deux–Sèvres. p.239. Paris: J.-B. Baillière & fils.

Savulescu T. 1952. Flore Republicii Populare Române. Bucharest: Editura Academiei.

Schaffner JH. 1912. New and rare plants of Ohio. *The Ohio Naturalist* 12:457.

Schnizlein A, Frickhinger A. 1848. *Die Vegetations–Verhältnisse der Jura– und Keuperformation in den Flussgebieten der Wörnitz und Altmühl. Mit einer geognostisch–topographischen Karte des Bezirkes*. p.76. Nördlingen: C.H. Beck.

Schönheit FCH. 1850. *Taschenbuch der Flora Thüringens*. Rudelstadt: L. Renovanz.

Schreiber P. 1916. Beiträge zur Flora des Zwittauer Gebietes. *Zeitschrift des Mährischen Landesmuseums* 15:67–95.

Schulz OE. 1911. Neue Beobachtungen im Kreise Zauch–Belsig. *Verhandlungen des Botanischen Vereins für die Provinz Brandenburg* 53: p.8

Scott EGF. 1896. *The flora of Dumfriesshire*. p.144. Dumfries: J. Maxwell & son.

Senghas K, Seybold S. 2003. *Flora von Deutschland und angrenzender Länder*. Wiebelsheim: Meyer Verlag GmbH & Co.

Sennikov A, Lazkov G, Naumenko A. 2014. New records in vascular plants alien to Kyrgyzstan. *Biodiversity Data Journal* 2, e1018. <http://dx.doi.org/10.3897/bdj.2.e1018>

Shuttleworth RJ. 1838. *Account of a Botanical Excursion in the Alps of the Canton of Valais, Switzerland, in August 1835 ; and Catalogue of the Plants collected, with occasional Remarks*. Magazine of zoology and botany 2:1–27.

Smith A. 1895. *A contribution to South African materia medica*. Capetown: Juta.

Smith JE. 1824. *The English flora*. London: Longman.

Solinger H. 1997. *Der Wiener Augarten und seine Flora*. p.206. H. Solinger.

Sommier S. 1903. Nuove Aggiunte Alla Florula di Giannutri. *Bullettino della Società Botanica Italiana.* 1903:228–232.

Sommier S, Gatto AC. 1912. Flora melitensis nova. Bollettino del R. *Orto botanico di Palermo. Nuova serie*. 11:1–502.

Sommier S. 1908. Le isole pelagie; Lampedusa, Linosa, Lampione e la loro flora con un elenco completo delle piante di Pantelleria. p.274. Firenze: Luigi Chiti.

Sommier S. 1922. Flora dell'isola di Pantelleria. Firenze: M. Ricci.

Souche B. 1893. Herborisation du 25 Juin 1893. *Bulletin de la Société botanique des Deux–sèvres* p. 57–60.

Spitzner W. 1888. Correspondenz. *Österreichische botanische Zeitschrift* 38:143–147.

Sprengel KPJ. 1832. *Curtii Sprengel Flora Halensis*. Halae: Sumtibus Kümmelii.

Stace C. 2010. *New Flora of the British Isles* (3rd ed.). Cambridge: Cambridge University Press.

Stewart R. 1860. *Handbook of the Torquay flora; comprising the flowering plants and ferns growing in and around Torquay, with their respective habitats*. p.107. Torquay: E. Croydon, Royal Library.

Stossich A. 1875. Catalogo delle piante dell'isola di S. Catterina presso Rovigno. *Bollettino della Società adriatica di scienze naturali in Trieste* 1:226–232.

Stowell HA. 1857. Faversham Plants. *The Phytologist* 2:153–156.

Tenore M. 1808. *Saggio sulle qualita medicinali delle piante della flora Napolitana*. p.119. Napoli: Nella Tipografia Coda.

Terracciano N. 1891. Synopsis plantarum vascularium Montis Pollini. *Annuario del R. Istituto botanico di Roma. Redatto dal Romualdo Pirrota*. 4.

Tessendorf F. 1906. Pflanzengeographie von Europa. *Just's botanischer jahresbericht. Systematisch geordnetes repertorium der botanischen literatur aller länder*. 34:449–684.

Tinant FA. 1836. *Flore luxembourgeoise*. p.140. Luxembourg: J. P. Kuborn.

Tomaschek A. 1859. *Flora der Umgebung von Lemberg*. 4. Wien.

Tommasini M, Marchesetti C. 1895. Flora Dell'Isola di Lussino. *Atti del museo civico di storia naturale di Trieste* 9: p.94

Tornabene F. 1891. *Flora Aetnea*. 3: p.294. Catinae: Francisci Galati.

Townsend Mackay J. 1836. *Flora hibernica: comprising the flowering plants, ferns, Characeæ, Musci, Hepaticæ, Lichenes and Algæ of Ireland : arranged according to the natural system with a synopsis of the genera according to the Linnæan system*. Dublin: William Curry jun. & company.

Traugott Bucher C. 1806. *Florae Dresdensis nomenclator*. p.55. Dresden,Waltherschen Hofbuchhandlung.

Trimen H, Thiselton-Dyer WH. 1869. *Flora of Middlesex*. London: R. Hardwicke.

Turland NJ, Chilton L, Press JR. 1993. *Flora of the Cretan Area*. London: HMSO.

Turner W. 1548. *The names of herbes. (with an introduction, an index of English names, and an identification of the plants enumerated by Turner. by James Britten*. London: Published for the English Dialect Society by N. Trübner 1881.

Uotila P. 1990. *Chenopodium* L. In: Castroviejo S, Laínz M, López Gonzáles G, Montserrat P, Muñoz Garmendia F, Paiva J & Villar L (eds.), Flora Iberica, Plantas vasculares de la Península Ibérica e Islas Baleares 2. Madrid: CSIC-CSIC Press: 484–500.

Urban I. 1881. Flora von Gross –Lichterfelde und Umgebung. *Verhandlungen des Botanischen Vereins für die Provinz Brandenburg* 22:26–57.

van de Bosch RB. 1850. *Prodromus Florae Batavae*. 1: p.221. Nijmegen: F.E. Macdonald.

Vanden Berghen C. 1981. Liste commentée des plantes vasculaires observées dans l'île de. Djerba (Tunisie méridionale). *Lejeunia* 105:38.

Van Rompaey E, Delvosalle L. 1972. *Atlas van de Belgische en Luxemburgse flora*. Brussels: Nationale plantentuin van België.

Verlot B. 1865. *Le guide du botaniste herborisant*. p.389. Paris: JB. Baillière et fils.

Vladimirov V, Dane F, Stevanovic V, Tan K. 2009. New floristic records in the Balkans: 12. *Phytologia Balcanica* 15(3):431–452.

von Dalla Torre KW, von Sarnthein L. 1909. Die Farn– und Blütenpflanzen (Pteridophyta et Siphonogama. von Tirol, Vorarlberg und Liechtenstein. p.964.

von Halácsy E. 1896. *Flora von Niederösterreich*. p.436. Prague: F. Tempsky.

von Halácsy E. 1910. Aufzählung der von Dr. B. Tuntas auf der Insel Scyros der nördlichen Sporaden im Juni 1908 gesammelten Arten. *Österreichische botanische Zeitschrift* 60:141–145.

von Hayek, A. 1908. Flora von Steiermark. Berlin: Borntraeger.

von Haller A. 1753. *Enumeratio plantarum horti regii et agri gottingensis*. p.21. Gottingen: A. Vandenhoeckii.

von Ledebour KF. 1844. Flora Rossica; sive, Enumeratio plantarum in totius Imperii Rossici provinciis Europaeis, Asiaticis et Americanis hucusque observatarum, auctore Carolo Friderico a Ledebour. Stuttgartiae: Sumptibus Librariae E. Schweizerbart.

Waga J. 1847. *Flora Polonica phanerogama*. 1: p.450. Varsaviae: Stanislai Straeski.

Walker R. 1833. *The flora of Oxfordshire and its contiguous counties*. Oxford: H. Slatter.

Watson W. 1912. Account of the Excursions (1910). *Transactions of the Edinburgh Field Naturalists' and Microscopical Society* 6: p.275.

Willdenow KL. 1787. *Florae Berolinensis prodromus*. p.98. Berlin: Wilhelmi Viewegii.

Willkomm M, Lange J. 1861. *Prodromus florae hispanicae, seu synopsis methodica omnium plantarum in Hispania*. Stuttgart: E. Schweizerbart.

Wimmer F. 1841. *Flora von Schlesien, preussischen und österreichischen Antheils, oder, Vom oberen Oder– und Weichsel–Quellen–Gebiet : nach natürlichen Familien, mit Hinweisung auf das Linnéische System*. p.84. Breslau: Ferdinand Hirt.

Wise JR, Crane W, Linton WJ. 1867. *The New Forest : its history and its scenery*. Cornhill: Smith, Elder and Co.

Withering W. 1845. *A systematic arrangement of British plants*. London: Adam Scott.

Wood R. 1883. Further additions to the list of flowering plants of Cumberland (West and East). *Transactions of the Cumberland Association for the Advancement of Literature and Science* 8: p.31.

Woodforde J. 1824‎. *A catalogue of the indigenous phenogamic plants, growing in the neighbourhood of Edinburgh*. London: Longman, Hurst, Rees, Orme, Brown.

Woodruffe–Peacock EA. 1898. Fossdyke Plants. *The Naturalist*. p.306.

Zajac M, Zajac A, Zemanek B. 2006. *Flora Cracoviensis Secunda*. p.66. Cracow: Jagiellonian University.

Zohary M. 1966. Equisetaceae to Moringaceae. *Flora Palaestina* 1: p.141.

Zumaglini AM. 1849. *Flora Pedemontana*. Torino: J. Favale.
