# Supplementary material for: Piecing together the biogeographic history of Chenopodium vulvaria L. using botanical literature and collections
Source: PeerJ. 2015 Jan 8;3:e723. doi: 10.7717/peerj.723 (PMC4304866; doi:10.7717/peerj.723)
Supplement: File S2 — Citations of data providers from the Global Biodiversity Information Facility and the Atlas of Living Australia. [file peerj-03-723-s002.docx]

1. Phytochorologie des départements français, http://data.gbif.org/datasets/resource/12442
2. Phanerogams herbarium specimens, http://data.gbif.org/datasets/resource/13686
3. Phanerogamic Botanical Collections (S), http://data.gbif.org/datasets/resource/8113
4. Botany (UPS), http://data.gbif.org/datasets/resource/1045
5. Missouri Botanical Garden, http://data.gbif.org/datasets/resource/12084
6. Fundación Biodiversidad, Real Jardín Botánico (CSIC): Anthos. Sistema de Información de las plantas de España, http://data.gbif.org/datasets/resource/9090
7. Lund Botanical Museum (LD), http://data.gbif.org/datasets/resource/1028
8. Herbario de la Universidad de Sevilla, SEV, http://data.gbif.org/datasets/resource/283
9. USDA PLANTS Database, http://data.gbif.org/datasets/resource/1066
10. Herbario de la Universidad de Salamanca: SALA, http://data.gbif.org/datasets/resource/239
11. Nationaal Herbarium Nederland, http://data.gbif.org/datasets/resource/11520
12. Inventaire National du Patrimoine Naturel (I084) : Flore Franco-Belge, http://data.gbif.org/datasets/resource/14299
13. Jardín Botánico Atlántico, Gijón: JBAG-Laínz, http://data.gbif.org/datasets/resource/8082
14. Database Schema for UC Davis [Herbarium Labels], http://data.gbif.org/datasets/resource/734
15. Herbario de la Universidad de Sevilla, SEV-Historico, http://data.gbif.org/datasets/resource/284
16. Herbarium Senckenbergianum Görlitz (GLM), http://data.gbif.org/datasets/resource/1105
17. Natural History Museum, Vienna - Herbarium W, http://data.gbif.org/datasets/resource/13042
18. SysTax - Botanical Gardens, http://data.gbif.org/datasets/resource/14438
19. EURISCO, The European Genetic Resources Search Catalogue, http://data.gbif.org/datasets/resource/1905
20. NMNH Botany Collections, http://data.gbif.org/datasets/resource/1874
21. inatura - Erlebnis Naturschau Dornbirn, http://data.gbif.org/datasets/resource/1866
22. Herbarium Senckenbergianum (FR), http://data.gbif.org/datasets/resource/8311
23. Universidad de Granada, Herbario: GDAC, http://data.gbif.org/datasets/resource/1742
24. Aranzadi Zientzi Elkartea, http://data.gbif.org/datasets/resource/248
25. Vascular Plant Collection - University of Washington Herbarium (WTU), http://data.gbif.org/datasets/resource/126
26. Herbier de Strasbourg, http://data.gbif.org/datasets/resource/12756
27. California State University, Chico, http://data.gbif.org/datasets/resource/737
28. Plantae, TAIF (Taiwan e-Learning and Digital Archives Program, TELDAP), http://data.gbif.org/datasets/resource/8053
29. Dpto de Botánica, Ecología y Fisiología Vegetal (herbario_cofc).Facultad de Ciencias.Universidad de Córdoba, http://data.gbif.org/datasets/resource/292
30. Universidad de Granada, Herbario: GDA, http://data.gbif.org/datasets/resource/1741
31. Observations du Conservatoire botanique national du Bassin parisien., http://data.gbif.org/datasets/resource/1103
32. Seed collection – Dead seeds for evaluation and observation purposes, http://data.gbif.org/datasets/resource/8334
33. Carnet en Ligne, http://data.gbif.org/datasets/resource/11503
34. NSW herbarium collection, http://data.gbif.org/datasets/resource/968
35. RBGE Herbarium (E), http://data.gbif.org/datasets/resource/8402
36. Herbarium specimens - Harmas de J. H. Fabre, http://data.gbif.org/datasets/resource/13679
37. WAHerb, http://data.gbif.org/datasets/resource/13675
38. University Museums of Norway (MUSIT), http://data.gbif.org/datasets/resource/1996
39. Institut Botanic de Barcelona, BC, http://data.gbif.org/datasets/resource/299
40. Tiroler Landesmuseum Ferdinandeum, http://data.gbif.org/datasets/resource/11971
41. SANT herbarium vascular plants collection, http://data.gbif.org/datasets/resource/222
42. Australian National Herbarium (CANB), http://data.gbif.org/datasets/resource/47
43. UCJEPS TAPIR Provider, <http://data.gbif.org/datasets/resource/1413>
44. Dataflos, http://data.gbif.org/datasets/resource/13502
45. Biological and palaeontological collection and observation data MNHNL, http://data.gbif.org/datasets/resource/8107
46. Leiner-Herbar Konstanz, http://data.gbif.org/datasets/resource/13545
47. Royal Museum of Central Africa - Metafro-Infosys - Prelude, http://data.gbif.org/datasets/resource/96
48. Herbarium specimens, http://data.gbif.org/datasets/resource/13685
49. Flora exsiccata Bavarica, http://data.gbif.org/datasets/resource/1092
50. Real Jardin Botanico (Madrid), Vascular Plant Herbarium (MA), http://data.gbif.org/datasets/resource/240
51. Botánica, Universidad de León: LEB-Cormo, http://data.gbif.org/datasets/resource/8003
52. Collection d'animaux marins et plantes du Museum d'Histoire Naturelle de Nice, http://data.gbif.org/datasets/resource/13733
53. Herbarium specimens, http://data.gbif.org/datasets/resource/13677
54. Fundación Carl Faust: Herbario del Jardí Botànic Marimurtra: HMIM, http://data.gbif.org/datasets/resource/12966
55. Herbarium Berolinense, http://data.gbif.org/datasets/resource/1095
56. Institut Botanic de Barcelona, BC-Histórico, http://data.gbif.org/datasets/resource/1523
57. Herbarium specimens, http://data.gbif.org/datasets/resource/13688
58. Herbarium WU, http://data.gbif.org/datasets/resource/1496
59. Herbarium (ALA), Alaska, http://data.gbif.org/datasets/resource/975
60. IPK Genebank, http://data.gbif.org/datasets/resource/1851
61. Botánica, Universidad de León: LEB, http://data.gbif.org/datasets/resource/11686
62. Herbarium specimens data, http://data.gbif.org/datasets/resource/13668
63. Escuela Técnica Superior de Ingenieros de Montes, UPM: EMMA, http://data.gbif.org/datasets/resource/278
64. Staatliches Museum für Naturkunde Stuttgart, Herbarium, http://data.gbif.org/datasets/resource/1100
65. New Zealand National Plant Herbarium (CHR), http://data.gbif.org/datasets/resource/474
66. Instituto de Botánica Darwinion, http://data.gbif.org/datasets/resource/13398
67. South Australia Flora Observations, http://data.gbif.org/datasets/resource/12710
68. Colección científica del Museo de Historia Natural Alfredo Dugés, http://data.gbif.org/datasets/resource/13368
69. Vascular Plant Herbarium, Trondheim (TRH), http://data.gbif.org/datasets/resource/7978
70. Consortium of California Herbaria, http://data.gbif.org/datasets/resource/9153
71. Impetus - Herbarium Hamburgense, http://data.gbif.org/datasets/resource/1605
72. Vascular Plant Herbarium, Oslo (O), http://data.gbif.org/datasets/resource/1078
73. Herbarium of Oskarshamn (OHN), http://data.gbif.org/datasets/resource/1024
74. Universidad de Málaga: MGC-Cormof, http://data.gbif.org/datasets/resource/8105
75. Herbarium (UNA), http://data.gbif.org/datasets/resource/775
76. The Himalayan Uplands Plant database (HUP Version 1), http://data.gbif.org/datasets/resource/14158
77. Israel Nature and Parks Authority, http://data.gbif.org/datasets/resource/1431
78. Jardín Botánico de Córdoba: Herbarium COA, http://data.gbif.org/datasets/resource/247
79. Cartografía de vegetación a escala de detalle 1:10.000 de la masa forestal de Andalucía, http://data.gbif.org/datasets/resource/10833
80. Herbario de la Universidad de Almeria, http://data.gbif.org/datasets/resource/244
81. Sistema de Información de la vegetación Ibérica y Macaronésica, http://data.gbif.org/datasets/resource/14072
82. Universidad de Extremadura, UNEX, http://data.gbif.org/datasets/resource/255
83. CIBIO, Alicante:ABH-GBIF, http://data.gbif.org/datasets/resource/251
84. Generalitat Valenciana. Banco de Datos de la Biodiversidad de la Comunitat Valenciana, http://data.gbif.org/datasets/resource/8004
85. Herbarium Willing, http://data.gbif.org/datasets/resource/1096
86. United States National Plant Germplasm System Collection, http://data.gbif.org/datasets/resource/1429
87. Hortus Botanicus Sollerensis Herbarium (FBonafè), http://data.gbif.org/datasets/resource/300
88. Vascular Plant Collection, http://data.gbif.org/datasets/resource/622
89. Catálogo Florístico Histórico de Navarra. Gobierno de Navarra, http://data.gbif.org/datasets/resource/12958
90. Herbario de la Universidad Pública de Navarra, Pamplona: UPNA-H, http://data.gbif.org/datasets/resource/8007
91. Botanical Information System of Geneva, http://data.gbif.org/datasets/resource/14176
92. Inventaire National du Patrimoine Naturel (ONF) : Données ONF faune-flore-fonge,
93. Inventaire National du Patrimoine Naturel (I062) : Flore de l'Ain, http://data.gbif.org/datasets/resource/14265
94. FlorKart - FlorenKartierung Gefaesspflanzen, http://data.gbif.org/datasets/resource/14519
95. Inventaire National du Patrimoine Naturel (I093) : Flore du Massif Armoricain, http://data.gbif.org/datasets/resource/14298
96. Inventaire National du Patrimoine Naturel (I067) : Flore d'Ille-et-Vilaine, http://data.gbif.org/datasets/resource/14293
97. Biologiezentrum Linz, http://data.gbif.org/datasets/resource/1104
98. Herbarium GJO, http://data.gbif.org/datasets/resource/1484
99. Perchtoldsdorfer Heide, http://data.gbif.org/datasets/resource/7863
100. Naturhistorisches Museum Mainz, Botanical Collection, http://data.gbif.org/datasets/resource/12677
101. Botanical Society of the British Isles - Vascular Plants Database, http://data.gbif.org/datasets/resource/839
102. Botanical Society of the British Isles - Vascular Plants Database additions since 2000, http://data.gbif.org/datasets/resource/11925
103. SINGER Coordinator, http://data.gbif.org/datasets/resource/8349
104. The System-wide Information Network for Genetic Resources (SINGER), http://data.gbif.org/datasets/resource/1430
105. Florabank1 - A grid-based database on vascular plant distribution in the northern part of Belgium (Flanders and the Brussels Capital region), http://data.gbif.org/datasets/resource/14149
106. Belgian IFBL Flora Checklists (1939-1971), http://data.gbif.org/datasets/resource/10969
107. Natural History Museum Maastricht (NL) - Herbarium, http://data.gbif.org/datasets/resource/14173
108. Dutch Vegetation Database (LVD), http://data.gbif.org/datasets/resource/12695
109. South East Wales Biodiversity Records Centre - CCW Regional Data : South East Wales Non-sensitive Species Records, http://data.gbif.org/datasets/resource/12702
110. Hertfordshire Natural History Society Flora Group - Hertfordshire Flora Survey Records 1987-2005, http://data.gbif.org/datasets/resource/11892
111. Suffolk Biological Records Centre - Suffolk Biological Records Centre (SBRC) dataset, http://data.gbif.org/datasets/resource/11927
112. Dr Francis Rose Field Notebook Project - Field Notebook Records of Dr Francis Rose 1950's to 1990's, http://data.gbif.org/datasets/resource/11887
113. Nijmegen National History Museum (NL) - Herbarium, http://data.gbif.org/datasets/resource/9185
114. The Flora of County Waterford, http://data.gbif.org/datasets/resource/10797
115. MfN - Fossil plants (Cenophytic), http://data.gbif.org/datasets/resource/9179
116. Vascular Plants, Field notes, Oslo (O), http://data.gbif.org/datasets/resource/1079
117. Herbarium, Botany Unit, Finnish Museum of Natural History (H), http://data.gbif.org/datasets/resource/14356
118. Herbarium, Centre for Biodiversity, University of Turku, Finland (TUR), http://data.gbif.org/datasets/resource/14064
119. Records provided by State Herbarium of South Australia, accessed through ALA website. http://collections.ala.org.au/public/show/co48
120. Department of Environment, Water and Natural Resources, Biological Databases of SA (BDSA), Date of Extraction: May 2010. http://collections.ala.org.au/public/show/dr366
121. Records provided by South Australia, Department of Environment, Water and Natural Resources, accessed through ALA website. http://collections.ala.org.au/public/show/dp32
122. Records provided by The Royal Botanic Gardens & Domain Trust, accessed through ALA website. http://collections.ala.org.au/public/show/in50
123. Records provided by Tasmanian Museum and Art Gallery, accessed through ALA website. http://collections.ala.org.au/public/show/in25
124. Records provided by Tasmanian Herbarium, accessed through ALA website. http://collections.ala.org.au/public/show/co60
125. Records provided by Australia's Virtual Herbarium, accessed through ALA website. http://collections.ala.org.au/public/show/dp36
126. Records provided by National Herbarium of New South Wales, accessed through ALA website. http://collections.ala.org.au/public/show/co54
127. Records provided by Australian National Herbarium, accessed through ALA website. http://collections.ala.org.au/public/show/co12
128. Records provided by Department of Environment and Natural Resources, accessed through ALA website. http://collections.ala.org.au/public/show/in41
129. Australia's Virtual Herbarium, a resource of the Council of Heads of Australasian Herbaria and its member Herbaria listed at www.chah.gov.au.
130. The Council of Heads of Australasian Herbaria (1999-) Australia's Virtual Herbarium www.chah.gov.au/avh [Accessed 25 February 2013]. http://collections.ala.org.au/public/show/dr376
131. Records provided by Royal Botanic Gardens Melbourne, accessed through ALA website. http://collections.ala.org.au/public/show/in21
132. Records provided by Centre for Australian National Biodiversity Research, accessed through ALA website. http://collections.ala.org.au/public/show/in5
133. Records provided by Western Australia, Department of Environment and Conservation, accessed through ALA website. http://collections.ala.org.au/public/show/in33
134. Records provided by Western Australian Herbarium, accessed through ALA website. http://collections.ala.org.au/public/show/co75
135. Records provided by National Herbarium of Victoria, accessed through ALA website. http://collections.ala.org.au/public/show/co55
