# Supplementary material for: Piecing together the biogeographic history of Chenopodium vulvaria L. using botanical literature and collections
Source: PeerJ. 2015 Jan 8;3:e723. doi: 10.7717/peerj.723 (PMC4304866; doi:10.7717/peerj.723)
Supplement: Table S3 — The words and phrases from specimens and Floras contributing to each broad habitat category. [file peerj-03-723-s003.docx]

| **animal waste**  pâturages fumés par le bétail; Düngerstätten; Dunghaufen; dunghill; dunghills; estercoleros; férteis; fumiers; hühnerhöfe; manure-heaps; Misthaufen; pinguibus; près des fumiers; proximidades de establos; Race Course; rangeerterrein; Stallungen; stercoratis; stickstoffreiche Ruderalstellen; ved Møddinger; von tierischen Flüssigkeiten oder von Menschen. | **boundaries**  ad muros; al piede dei muri; along fences; along walls; an der Stadtmauer; an Mauern; Au pied des murs; au pied d'un mur; bords des murs; entre los paredones; foot of the church yard wall; foot of the city walls; foot of walls; Hecken; keritesei; le long d´un mur; le long des haies; le long des murs; mauern; mud walls; muren; muris; paling; palings; Pied de murs; pied des murs; près des murs; prope muros; sepes; under a wall; under walls; voet oude murs; walles; zäune; Zäunen; zdi. | **wetland**  alkaline river banks; aluviones; bottom of sayrs; cunetas; encharcados o pisoteados; fossés; fossis; húmidos; in gutter; lake coasts; lugares temporalmente encharcados; on canal bank; ravine; Secus fluvium; vores riera. | **habitation**  An bebauten orten; an Häusern; bei Ortschaften; città; Dörfer; Dorfern; Dörfern; falvak; Häusern; houses; inhabited areas; nomad camp sites; Orte; Ortes; Ortschaften; près des habitations; Stadt; Städte; Städten; suburban; suburbs; town; towns; um Stadten; urbis; varosok; village; Villages; voisinage des habitations; vorstadt. |
| --- | --- | --- | --- |
| **dry & bare soil**  bare; bare patches; barish places; dry; dry banks; exsiccatis; Rydde Pladser; terra sotto gli alberi; Trockene. | **industry**  Bij de glasblazerijte; factories; graanafval; graanoverslagbedrijf; mills. | **hills**  in collibus ad; les collines; poggi. | **shipping**  ballast; Ballast ground; hafen; ports. |
| **disturbed and grazed**  campis; campos; camps; camps incultis; disturbed places; fallow field; fields; grassy slopes; in olitoriis vulgare; incultis; Parlagokon; ruigte; ruigterrein; sur les champ de foire; vuig terrein. | **rail**  abords de la gare; Eisenbahndämmen; railway bridge near the station; stellen auf bahnhöfen; talus du chemin de fer; tegenover het station. | **coastal**  by the Channel; by the sea; cliffs; coastal habitats; dunes; Felsküste; near sea; near the sea; nieuwe zeedijk; région maritime; rocky cliff; salt-marshes; seashores; the seaside. | **sand and rock** gravelly ground; kleiig zand; Links; rocky; Sables; sand; sandy; shingle beaches; Terrenos pedregosos. |

| **horticulture**  Lieux cultivés; agris; allotment fields; allotments; bebauten Stellen; bouwlanden; Champs cultivés; container van olijf; cultis; cultivated fields; cultivated ground; cultures; dans les lieux cultivé; dans un vase; field; fields; garden; garden ground; gardens; gardine; Gartenlande; Hort de la Font; hortis; huertas; in agris cultis; in cultis; in fields; in garden; in locis cultis; irrigated; irrigated plants; Jardin; jardines; jardins; Küchengärten; les cultures; les jardins; lieux cultives; Lieux cultivés; marges de conreus; oleraceis; Orchards; Paa dyrkede Steder; Plantation; terres cultivées; Versuchsfeld; weed in field. | **waste**  aggeres; aggeribus; aschhoopen; Auf Schutt; auf Schütt; auf Schuttst; auf wüsten Plätzen; décombres; encombries; entulhos; escombros; fra macerie; friches aux camions; In ruderatis; In ruderatis prope; les décombres; Lieux incultes; llocos incultes; los escombros; Luoghi ruderali; nos entulhos; Ödland; on rubbish; paper-tip; puin; rubbish; rubbish tip; rubbishy places; rubbishy places; rubble; Ruderal; Ruderal places; rudéralisés; Ruderalstellen; ruderatis; ruderum acervis; Ruins; Schutt; Schutthaufen; schuttplätze; Schuttplätzen; stortplaats; stortterrein; terrain vague; terrains vagues; tips; vuilnishoopen; Vuilsort; vuilstort; waste ground; Waste grounds; waste places; wüste Plätze; wüsten Plätzen; wüsten Stellen. | **roads**  ad vias; Ai margini delle vie; along streets; along the roads; an Straßen; An Wegen; Auf Strassen; beira dos caminhos; bords des chemin; bords des chemins; Bords des Routes; by the side of tracks; cattle trails; cobbled streets; crack between base of brick wall and tarmac road; Grünstreifen zwischen den Fahrbahnen; le vie; Lungo le strade; lungo le vie; margini delle vie; orilla de la caminos; orilla de los caminos; plateas; ritze zwischen hauswand und Gehsteig; roads; roadside; roadsides; road-sides; routes; strade; strassen; Straßen; Strassenrand; street; sur le Boulevard; tussen straatstenen; utczain; vias; viis; wayside; Wege; Wegen; Wegränder. |  |
| --- | --- | --- | --- |
