# Supplementary figures and images for: Piecing together the biogeographic history of Chenopodium vulvaria L. using botanical literature and collections
Source: PeerJ. 2015 Jan 8;3:e723. doi: 10.7717/peerj.723 (PMC4304866; doi:10.7717/peerj.723)

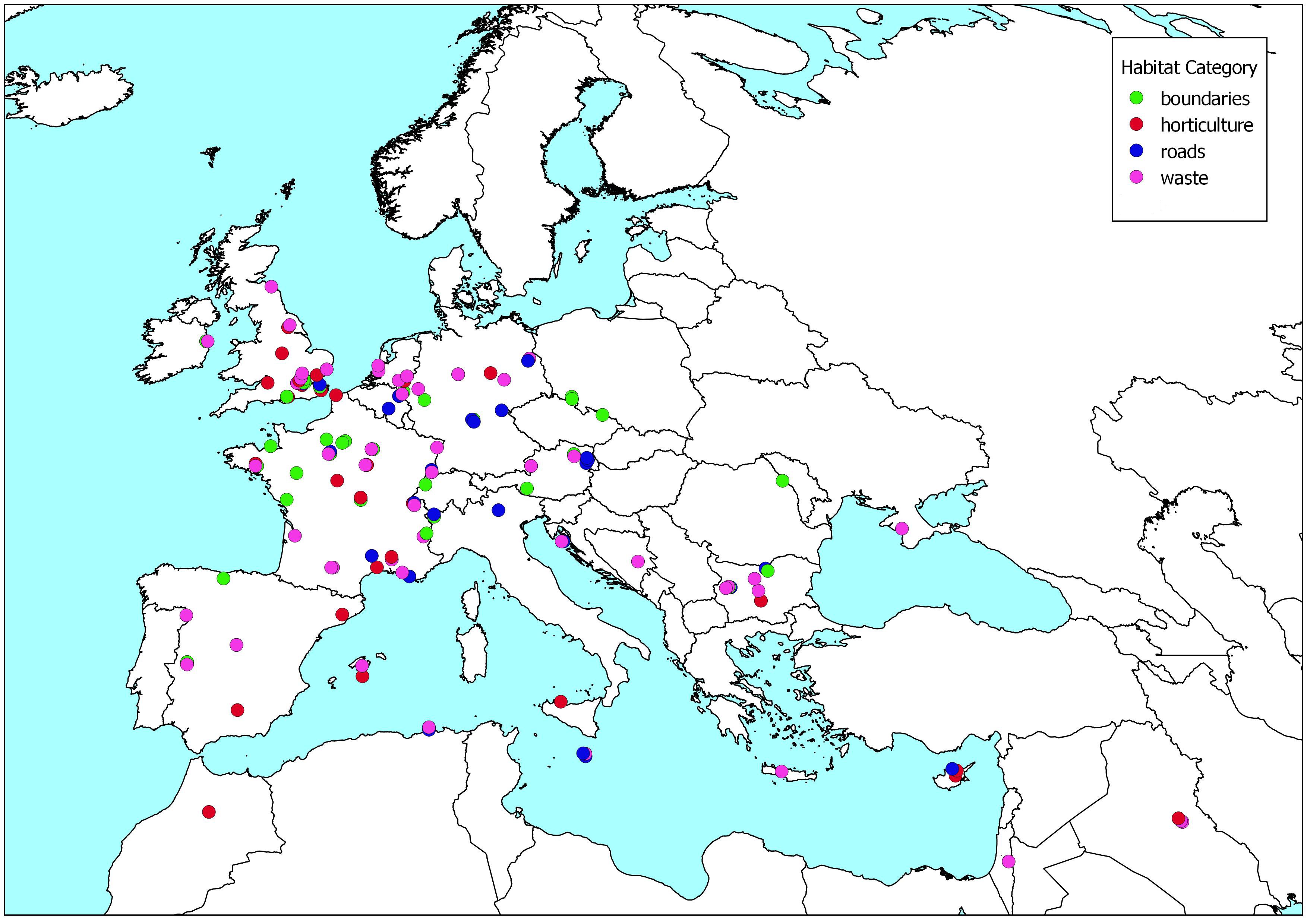

Supplement: Figure S4 — For the four most common habitat categories where specimens or observations had both a habitat category and a geolocation they have been mapped to show the distribution of habitat categories within Europe. Points have been randomly jittered to ensure overlapping points are visible. The map uses a Mollweide equal area projection. [file peerj-03-723-s004.jpeg]
